# Aging with AI companionship: the role of artificial intelligence in enhancing the mental wellbeing of older adults

**DOI:** 10.3389/fpubh.2026.1725763

**Published:** 2026-05-01

**Authors:** Yonggang Wang, Huanchen Tang, Jingchun Zhang, Yubo Wang, Xiaodong Liu

**Affiliations:** College of Fashion and Design, Donghua University, Shanghai, China

**Keywords:** artificial intelligence, mental wellbeing, older adults, self-determination theory, technology acceptance model

## Abstract

**Introduction:**

With the intensification of global population aging, mental health problems among older adults have become increasingly prominent. As an important innovative approach in smart eldercare, artificial intelligence (AI) has been gradually applied to older populations due to its advantages in emotional support, cognitive stimulation, and social interaction. However, there is still a lack of systematic empirical evidence regarding the mechanisms through which AI affects older adults’ mental wellbeing.

**Methods:**

Drawing on Self-Determination Theory and the Technology Acceptance Model, this study constructs a five-dimensional analytical framework consisting of autonomy, perceived ease of use, perceived usefulness, competence, and relatedness, and combines structural equation modeling (SEM) with fuzzy-set qualitative comparative analysis (fsQCA). Using survey data from 418 Chinese older adults aged 60 and above, we systematically explore the pathways through which AI influences their mental wellbeing.

**Results:**

The findings indicate that autonomy exerts the most significant positive effect on older adults’ mental wellbeing, while perceived ease of use, perceived usefulness, competence, and relatedness also have positive impacts.

**Discussion:**

By granting older adults greater choice and decision-making power, AI enhances their sense of control over life and self-efficacy, thereby effectively promoting mental wellbeing. At the same time, the ease of use and practicality of AI technologies lower the threshold for adoption, increasing older adults’ intention to use and satisfaction. Furthermore, AI supports cognitive training, health management, and emotional communication, helping older adults maintain cognitive vitality, strengthen self-care abilities, and receive emotional support and social connection, which in turn further enhance their sense of wellbeing and quality of life. This study not only enriches the theoretical foundation of research on AI and mental wellbeing among older adults, but also provides empirical evidence for the optimization, design, and promotion of related technologies.

## 1 Introduction

With the accelerating process of global population aging, the health and wellbeing of older adults have increasingly become a central concern of society. According to the United Nations report World Population Prospects 2023, the global population aged 60 and above has already exceeded 1 billion and is projected to reach 2.1 billion by 2050 (1). China, as the country with the largest older population in the world, had 280 million people aged 60 and above in 2022, accounting for 19.8% of its total population (2). Against the backdrop of an aging society, how to improve older adults’ quality of life and promote their physical and mental health has become a pressing social issue. For a long time, the mental health of older adults has been relatively neglected. Studies have shown that, with advancing age, older adults are exposed to multiple stressors, including physical decline, an increasing burden of chronic diseases, changes in social roles, the death of relatives and friends, and separation from children, which make them prone to loneliness, depression, anxiety, and other negative emotions (3). Mental health not only affects older adults’ subjective quality of life, but is also closely related to their physical health status, adherence to disease management, and social adaptability (4). Therefore, while focusing on disease prevention and control as well as functional maintenance, it is of great theoretical and practical significance to explore effective psychological intervention approaches and systematically enhance the overall mental wellbeing of older adults.

In recent years, the rapid development of artificial intelligence (AI) technologies has created new possibilities for reshaping smart eldercare and geriatric care service systems (5). As a key technological pillar of smart eldercare, AI is gradually being embedded into older adults’ daily lives and into healthcare-related workflows (6). In terms of application scope, AI not only encompasses companionship-oriented products targeting emotional and social interaction—such as smart speakers (7), companion robots (8), and virtual assistants (9)—but is also widely used in structured health information collection (10), remote monitoring (11), risk warning (12), delivery of personalized intervention recommendations (13), and clinical decision support (14) within health management and care processes. For example, systems based on multimodal data and natural language processing can assist in the standardized collection of older adults’ medical histories and lifestyle information, improving the completeness and consistency of information records, and, on this basis, provide intelligent support for medical and nursing decisions, thereby optimizing geriatric care workflows (15). At the terminal service level for older users, AI can also provide integrated support for older adults’ multidimensional needs in the physical, psychological, and social domains through diversified functions such as health management (16), medication and daily-life reminders (17), rehabilitation training guidance (18), and affective interaction (19). Compared with traditional interpersonal companionship and social support, AI has advantages such as round-the-clock availability, timely response, a high degree of personalization, and low marginal cost. To a certain extent, it can compensate for deficiencies in older adults’ social support systems, help alleviate the pressure on care resources, and offer a new technological pathway for reducing older adults’ loneliness and psychological stress and enhancing their mental wellbeing.

Existing studies have mainly focused on the application of artificial intelligence in health management (16), daily care (20), and emergency assistance (21) for older adults, while systematic research on how AI affects their mental wellbeing remains relatively scarce. Some scholars have pointed out that AI can enhance older adults’ subjective wellbeing and life satisfaction through emotional interaction, information support, entertainment, and leisure (22). However, most of the current research is dominated by qualitative analyses and lacks empirical tests based on theoretical models. The mechanisms and underlying pathways through which AI influences older adults’ mental wellbeing have not been thoroughly explored. In addition, the impact of individual difference factors—such as older adults’ acceptance of AI, user experience, and level of technological literacy—on mental wellbeing also requires further investigation.

Anchored in the Technology Acceptance Model (TAM) and Self-Determination Theory (SDT), this study systematically examines the mechanisms by which AI enhances the mental wellbeing of older adults. The Technology Acceptance Model emphasizes that individuals’ perceived usefulness and perceived ease of use of new technologies significantly influence their adoption behaviors, whereas Self-Determination Theory focuses on how the satisfaction of three basic psychological needs—autonomy, competence, and relatedness—affects individuals’ motivation and psychological wellbeing. Integrating these two perspectives helps to comprehensively understand how AI improves older adults’ mental wellbeing, on the one hand by enhancing their technology acceptance, and on the other by satisfying their basic psychological needs. The innovations of this study are mainly reflected in the following aspects: First, this paper organically integrates TAM and SDT to construct a theoretical analytical framework of “artificial intelligence–technology acceptance–psychological needs–mental wellbeing,” thereby systematically revealing the multidimensional pathways through which AI affects older adults’ mental wellbeing. Second, this paper employs structural equation modeling (SEM) together with fsQCA to empirically analyze the relationships among variables including AI, technology acceptance, autonomy, competence, relatedness, and mental wellbeing. Through large-sample questionnaire surveys and multidimensional scale measurements, it overcomes the limitations of previous studies such as small sample sizes, single variables, and simplistic analytical methods, thus enhancing the scientific rigor and reliability of the conclusions. Third, taking into account the actual developmental status of smart eldercare in China, this study selects representative smart eldercare apps and platforms as research objects, and proposes targeted policy recommendations and product optimization pathways, providing theoretical support and practical references for governments, enterprises, and social organizations to promote innovation in smart eldercare services.

Based on the above analysis, this paper aims to address the following research questions: (1) To what extent can artificial intelligence enhance the mental wellbeing of older adults? (2) How do factors such as technology acceptance and satisfaction of psychological needs influence the relationship between AI and the mental wellbeing of older adults? (3) How can AI products and services be optimized to improve older adults’ acceptance of and user experience with intelligent technologies? By systematically examining these questions, this study seeks to provide theoretical support and practical guidance for the innovative development of smart eldercare services, to enable AI technologies to better serve the mental wellbeing of older adults, and to contribute to the construction of a positive, healthy, and fulfilling aging society.

## 2 Literature review and research hypotheses

### 2.1 Applications of artificial intelligence in accompanying older adults

With the intensification of global aging, the application of artificial intelligence (AI) technology in the field of older adult care has gradually become a focal point of attention in both academia and industry. In recent years, AI technology has demonstrated tremendous potential not only in health management and daily care but also in providing emotional support and promoting mental health (23). The main forms of AI companionship for older adults include social robots, virtual assistants, and smart speakers. Social robots can interact with older adults through voice, facial expressions, and movements, offering emotional comfort and daily companionship (24). Virtual assistants and smart speakers, utilizing voice recognition and natural language processing technologies, assist older adults in managing daily tasks, health monitoring, and information retrieval (25). These AI products not only possess basic life-assistance functions but are also gradually evolving towards diversified directions such as emotional interaction, cognitive training, and entertainment, thereby meeting the multi-level needs of older adults. Wada et al. conducted a long-term experiment on robot-assisted activities for older adults in a health service institution and found that robots could enhance social connections and reduce the occurrence of negative emotions through daily conversations and interactions (23). In addition, AI has also been shown to help improve cognitive function and social participation among older adults. Graham et al., in a comparative analysis, pointed out that AI not only provides emotional support but can also delay cognitive decline and promote active aging through cognitive training and recreational activities (26). Furthermore, AI can, to some extent, compensate for the shortcomings of traditional social support. Moyle et al. noted that AI can provide emotional comfort for older adults who live alone or are disabled, and can also enhance their self-efficacy and sense of social belonging through informational support and health reminders (27).

Despite the substantial achievements of existing research, there remain profound theoretical and methodological limitations. Most studies have simplified “companionship” to “dialogue” or “interaction,” neglecting the deeper psychological significance inherent in high-quality companionship. According to Self-Determination Theory (SDT), high-quality interpersonal relationships can nurture individuals’ needs for autonomy, competence, and relatedness. To what extent, then, can AI, as a non-human form of “companionship,” simulate or substitute these key factors? This is a question that urgently requires answers from the perspective of fundamental psychological theories. This study will move beyond simply addressing whether “AI is useful,” and instead aims to systematically answer the more fundamental scientific question of “how and why AI works” by integrating TAM and SDT to elucidate the process from “technology use” to “psychological benefit.”

### 2.2 Mental health in older adults

With the accelerating global population ageing, mental health problems among older adults have become a major public health challenge. According to data from the World Health Organization (WHO) and related epidemiological studies, depression and anxiety are the most prevalent mental disorders in later life. Volkert’s research indicates that mental health problems in older adults frequently co-occur with physical illnesses, but are often misattributed to normal ageing, leading to substantial underdiagnosis (28). Casey et al. emphasize that late-life depression not only increases the risk of suicide but also significantly accelerates cognitive decline and the onset and progression of Alzheimer’s disease (29). When examining the social determinants of mental health in older adults, social isolation and loneliness are among the most intensively discussed themes in the current literature. Holt-Lunstad et al. report that the impact of lacking social connections on mortality is comparable to that of smoking and obesity (30). Focusing specifically on mental health, Santini et al., drawing on data from tens of thousands of older adults, identify social isolation as an independent predictor of late-life depression and anxiety (31). Their findings suggest that, even after adjusting for socioeconomic status and baseline health, reductions in social interaction directly contribute to declines in mental health. Furthermore, the systematic review by Courtin and Knapp distinguishes between “objective isolation” and “subjective loneliness,” and points out that the detrimental impact of subjective loneliness on mental health often exceeds that of objectively living alone. This implies that future interventions should prioritize meeting older adults’ emotional needs rather than merely increasing the frequency of social contact (32).

In recent years, with the advancement of technology, the impact of the digital divide and digital inclusion on older adults’ mental health has attracted increasing attention. Lam et al. pointed out that policies aimed at improving the mental health of older adults should encourage internet use, especially as a tool to support communication (33). However, Cotten et al. also issued a caution that the use of technology must take into account older adults’ cognitive load, as poorly designed digital products may instead exacerbate their technology-related anxiety (34). In this context, and given that prior literature discussing “mental health” has often focused on clinical or negative indicators such as depression and loneliness, this study adopts a perspective grounded in positive psychology. Considering that the core objective of artificial intelligence technologies is not merely to eliminate negative emotions among older adults, but more importantly to create positive subjective experiences (such as pleasure and freedom from worry), this study explicitly selects “mental wellbeing” rather than the broader term “mental health” as the key outcome variable. The aim of this paper is to explore in depth the specific mechanisms and effectiveness of AI in enhancing older adults’ mental wellbeing, so as to provide precise theoretical foundations for promoting a shift in smart eldercare from “disease prevention” to “wellbeing enhancement.”

### 2.3 Technology acceptance model (TAM)

Since its introduction by Davis in 1989, the Technology Acceptance Model (TAM) has become a classic theoretical framework for explaining and predicting users’ adoption behaviors toward emerging information technologies. TAM emphasizes that “perceived usefulness” (PU) and “perceived ease of use” (PEOU) are the core variables influencing users’ willingness to adopt new technologies. In recent years, with the rapid development of artificial intelligence and smart older adult care technologies, TAM has been widely applied to studies on older adults’ acceptance and use of AI products, and has been continuously expanded and refined (35, 36).

In the field of smart older adult care, TAM provides a theoretical foundation for understanding older adults’ acceptance behaviors toward new technologies such as AI companionship, smart health devices, and smart older adult care platforms. Numerous empirical studies have shown that perceived usefulness and perceived ease of use significantly influence older adults’ willingness to adopt intelligent technologies. For example, Zhou et al. conducted an in-depth exploration of the behavioral intentions of older adult users in smart home services (37). Li et al. investigated the mechanisms by which older adults in retirement communities adopt smart health services, and their findings offer theoretical support for adoption behaviors and provide research evidence for understanding the needs of older adults regarding smart health services (35). TAM not only helps explain the adoption behaviors of older adults toward AI technologies but also provides theoretical support for understanding the mechanisms by which technology adoption affects mental health. Existing research has indicated that positive acceptance of AI technologies by older adults can enhance their social participation and self-efficacy, thereby improving their mental health (38). An et al. examined the structural relationship between subjective wellbeing and perceived usefulness in the willingness of older adults to use digital public services, aiming to enhance the wellbeing of older adults enjoying digital public services (39).

In summary, TAM provides a solid theoretical foundation for research on technology adoption in the field of smart older adult care. This paper systematically analyzes the mechanisms by which AI technologies affect the mental wellbeing of older adults, offering more scientific theoretical guidance and practical pathways for the innovation of smart older adult care services and the improvement of older adults’ wellbeing.

### 2.4 Self-determination theory (SDT)

Self-Determination Theory (SDT), proposed by Deci and Ryan, is one of the most important contemporary theories for explaining human motivation, behavior, and psychological health. SDT emphasizes that the satisfaction of three basic psychological needs—autonomy, competence, and relatedness—is key to fostering intrinsic motivation and enhancing wellbeing and mental health (40). A large body of empirical research has shown that if intelligent technologies and digital services can enhance older adults’ sense of autonomy, competence, and relatedness, they are more likely to be accepted and continuously used by this population (41). AI products, through personalized settings and interactions, enhance older adults’ experiences of autonomy (42). In addition, the ease of use and feedback mechanisms of intelligent technologies can improve older adults’ sense of competence, reducing technology-related anxiety and feelings of helplessness (43). More importantly, AI promotes connections between older adults and the outside world through emotional interaction, social functions, and informational support, thereby meeting their need for relatedness, effectively alleviating loneliness, and improving mental wellbeing (44). Khosravi et al. found that AI robots, in both institutional and home care settings, can significantly enhance older adults’ social participation and emotional connections through daily conversations, recreational activities, and health reminders, thereby improving their mental health (45).

In summary, although SDT has yielded abundant results in the fields of mental health and technology adoption among older adults, most existing research has focused on Western countries, and empirical studies on the compatibility between the psychological needs of older adults and AI technologies in Eastern cultural contexts such as China remain limited. Therefore, this paper integrates SDT with the Technology Acceptance Model (TAM) and other theoretical frameworks to systematically analyze the mechanisms by which AI affects the mental wellbeing of older adults.

### 2.5 Research hypotheses

**Figure 1:**
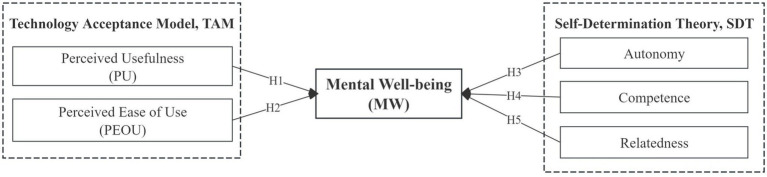
Schematic diagram of the research hypothesis model.

To systematically explore how artificial intelligence technology influences the mental wellbeing of older adults through different mechanisms, this study introduces the Technology Acceptance Model (TAM) and Self-Determination Theory (SDT) as its theoretical foundations. The TAM emphasizes that perceived ease of use and perceived usefulness are key prerequisites for promoting the adoption and continued use of AI technologies among older adults. These factors not only affect technology adoption behaviors but may also contribute to improvements in mental wellbeing. Meanwhile, SDT focuses on the role of AI technologies in satisfying older adults’ basic psychological needs for autonomy, competence, and relatedness, highlighting that the fulfillment of these needs is a core pathway for stimulating intrinsic motivation and enhancing mental health. Therefore, from the theoretical perspectives of TAM and SDT, this study aims to bridge the gap between “why to use” and “how use works,” systematically proposing and testing the mechanisms and pathways through which AI technologies affect the mental wellbeing of older adults. The specific research hypotheses are illustrated in Figure 1.

#### 2.5.1 The impact of the technology acceptance model (TAM) on the mental wellbeing of older adults

Perceived ease of use refers to the extent to which older adults find it easy to learn and operate artificial intelligence technologies (such as smart speakers and companion robots). Existing research indicates that the ease of use of technology can significantly reduce technology-related anxiety and resistance among older adults, thereby enhancing their confidence and willingness to use such technologies (46). In an empirical study on the adoption of smart health devices among Chinese older adults, Li et al. found that perceived ease of use not only directly influences older adults’ willingness to adopt but also indirectly promotes a positive attitude toward new technologies by enhancing self-efficacy (47). For older adults, AI companion products that are easy to operate, user-friendly, and offer natural interactions can effectively lower the learning threshold, making them more willing to try and continue using these technologies, thus laying a foundation for subsequent mental health promotion. Secondly, perceived usefulness emphasizes older adults’ subjective evaluation of the actual value of AI technologies, including aspects such as information acquisition, health management, entertainment, and emotional comfort. Relevant studies have shown that only when older adults truly feel that AI technologies can meet their life needs and improve their quality of life will they develop intrinsic motivation for continued use (48). Schroeder et al. pointed out that perceived usefulness not only directly affects the adoption behavior of older adults but also indirectly promotes improvements in their mental wellbeing by enhancing their sense of control over life and social participation (46).

The two core variables of the TAM not only influence older adults’ willingness to adopt AI technologies but may also affect their mental wellbeing through a series of psychological mechanisms. First, AI products that are easy to use and useful can enhance older adults’ self-efficacy and life satisfaction, reducing frustration and loneliness caused by technological barriers. Second, continued use of AI companion technologies helps older adults obtain more social support and emotional communication, thereby alleviating negative emotions such as depression and anxiety. Therefore, perceived ease of use and perceived usefulness are not only key factors driving older adults to “try” and “continue using” AI technologies, but also important antecedents influencing their mental wellbeing. Based on the above theoretical and empirical foundations, this study proposes the following hypotheses:

*H1*: The perceived ease of use of AI technologies positively influences the mental wellbeing of older adults.

*H2*: The perceived usefulness of AI technologies positively influences the mental wellbeing of older adults.

#### 2.5.2 The impact of self-determination theory (SDT) on the mental wellbeing of older adults

Self-Determination Theory (SDT) emphasizes that the satisfaction of three basic psychological needs—autonomy, competence, and relatedness—is the core mechanism for promoting intrinsic motivation, enhancing wellbeing, and improving mental health (40). In recent years, SDT has been widely applied in the fields of health promotion, technology adoption, and psychological intervention among older adults, and has shown unique value, especially in research on the impact of intelligent technologies on the mental health of older adults (45, 49).

First, autonomy support refers to older adults’ perception that AI enhances their sense of control and choice over their lives and environment. Seifert et al. found that smart home and AI devices, through voice commands and personalized settings, enable older adults to independently manage their daily lives, thereby strengthening their sense of control (41). This experience of autonomy not only boosts self-esteem and positivity among older adults but also effectively alleviates feelings of helplessness caused by role loss and dependence on others, thus promoting mental wellbeing. Second, competence support emphasizes that through interaction with AI technologies, older adults can successfully complete tasks, learn new knowledge, or solve problems, thereby feeling capable. After successfully using AI, older adults often experience a positive sense of “I can do it,” and this sense of competence helps counteract feelings of helplessness and self-doubt associated with aging, enhancing their self-efficacy and life satisfaction (50). Third, relatedness support focuses on the role of AI technologies in promoting social connections and emotional companionship for older adults. AI products, through daily conversations, emotional greetings, and social functions, can to some extent simulate interpersonal interactions and meet older adults’ needs for belonging and connection (51). AI can not only alleviate the loneliness of older adults living alone (52) but also enhance their social participation and wellbeing through informational support and emotional communication.

SDT posits that the satisfaction of these three psychological needs not only increases older adults’ willingness to continue using AI technologies but also significantly improves their mental health by enhancing intrinsic motivation and positive emotions. Based on the above theoretical and empirical foundations, this study proposes the following hypotheses:

*H3*: The support of autonomy provided by AI technologies positively influences the mental wellbeing of older adults.

*H4*: The support of competence provided by AI technologies positively influences the mental wellbeing of older adults.

*H5*: The support of relatedness provided by AI technologies positively influences the mental wellbeing of older adults.

## 3 Research methodology and data collection

### 3.1 Data inclusion criteria and research methods

**Figure 2:**
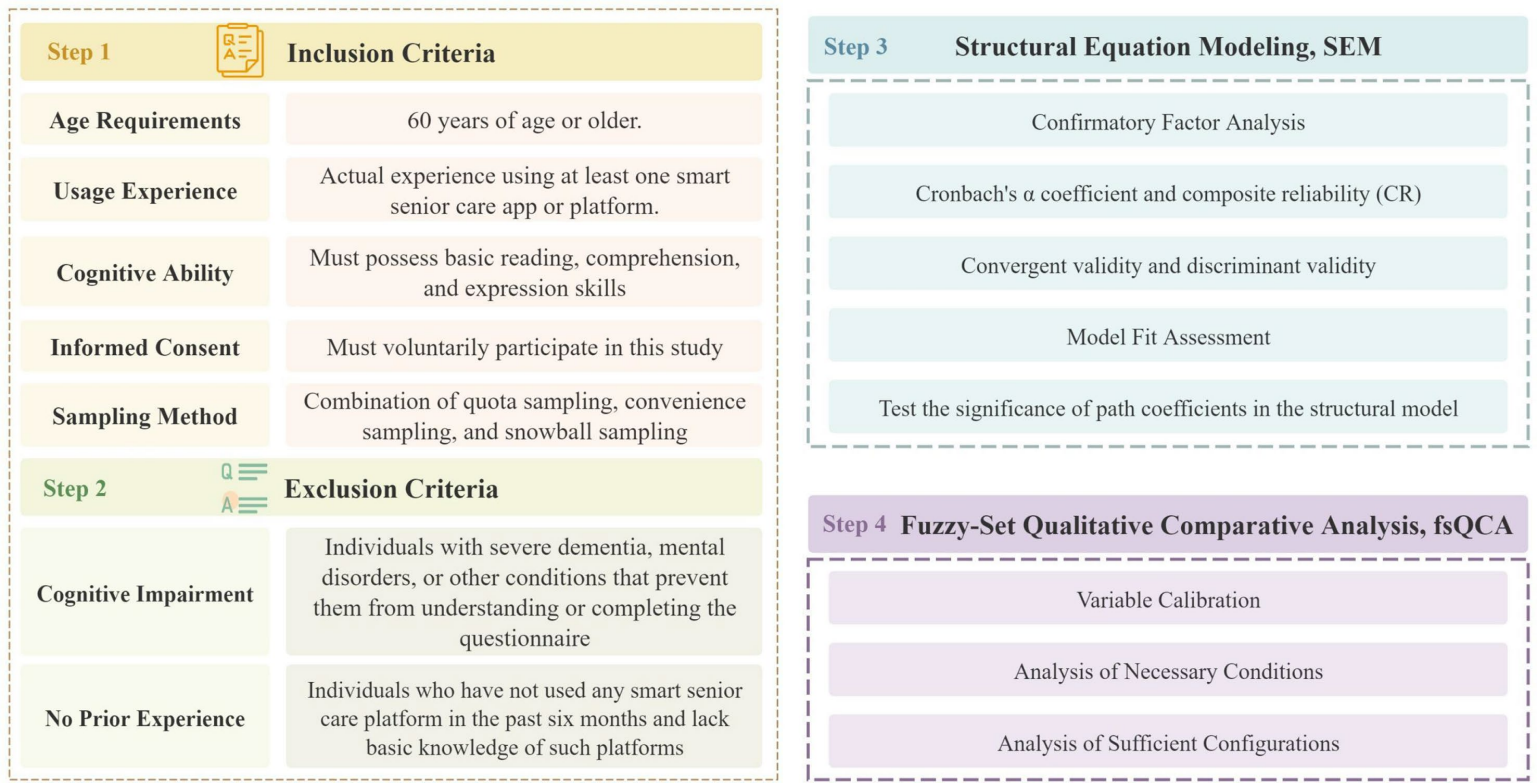
Data screening and research flowchart.

The target population of this study comprised older adults aged 60 years and above residing in mainland China who were currently using, or had the potential to use, smart eldercare platforms. To ensure diversity and representativeness of the sample, the definition of the target population and the recruitment process were refined as shown in Figure 2. In this study, the term “smart eldercare platforms and related technologies” primarily refers to the following types of Internet- and AI-based terminals and applications:

1. Voice-interactive smart devices, such as Xiaodu smart speakers and Tmall Genie. These devices typically integrate functions including voice assistants, information queries, scheduling and medication reminders, music and audiobook playback, delivery of basic health information, family voice or video calls, and partial smart home control.
2. Comprehensive eldercare service apps or platforms targeting older adults, such as the JD Health · JD Eldercare mobile application or mini-programs. Such platforms generally provide modules for health record management, online consultation and telemedicine, medication management and e-pharmacy services, physical examination and home-visit service appointments, linkage with home- and community-based eldercare services, wellness activities, and dissemination of health education content.
3. Smart health devices that can be connected to the aforementioned platforms, such as smart bands/watches, blood pressure monitors, and glucometers. These devices enable daily vital-sign monitoring, data upload, and abnormal-event alerts, with data viewed and managed through the platform interface.

It should be noted that this study focuses on the relatively integrated “smart eldercare services” and the associated interaction experiences that underlie these devices and applications, rather than on any single piece of hardware or a particular product with a fixed interface. Participants might use smart speakers of different brands, an eldercare service app, or a combination of both. Accordingly, in the questionnaire and interviews, we collectively refer to these various terminals and their associated services as “smart eldercare platforms” or “smart eldercare products.”

Participants were not required to use all functional modules of a platform. The inclusion criterion only required that, within the past 6 months, respondents had actually used at least one smart eldercare–related device or platform and had experienced several typical functions during use (e.g., health management, medication/healthcare reminders, daily living assistance, emotional companionship, social communication). Depending on their abilities and needs, different respondents might have used only a subset of functions—for example, some primarily used voice queries and entertainment playback, whereas others focused on health monitoring, medication reminders, or online consultations. This study examines older adults’ perceptions of their “overall user experience with smart eldercare technologies,” rather than evaluating any single product or a complete function package. On this basis, the sample inclusion criteria for this study were as follows: (1) Age ≥ 60 years; (2) Possession of basic reading and comprehension skills, and ability to complete the questionnaire independently or with minimal assistance; (3) Actual use within the past 6 months of at least one of the aforementioned types of smart eldercare devices or platforms (e.g., Xiaodu smart speaker, Tmall Genie, JD Health · JD Eldercare, or similar eldercare apps/products); (4) Voluntary participation with signed informed consent.

This study aims to systematically reveal the mechanisms by which artificial intelligence technology enhances the mental wellbeing of older adults. Given that these mechanisms involve not only the net effects among variables but also potentially complex causal pathways with multiple concurrent factors, a single research method is insufficient to fully capture this complexity. Therefore, this study innovatively adopts a mixed-methods research design that combines Structural Equation Modeling (SEM) and Fuzzy-Set Qualitative Comparative Analysis (fsQCA). First, IBM SPSS Statistics 27.0 and AMOS 26.0 software will be used to conduct SEM analysis to test the causal relationships among variables in the theoretical model. Subsequently, based on these results, fsQCA 3.0 software will be employed for configurational analysis to explore various antecedent “configurations” that lead to high levels of mental wellbeing. By leveraging the complementary strengths of the symmetric testing in AMOS and the asymmetric analysis in fsQCA, this study constructs a comprehensive panorama of the mechanisms by which AI technology enhances the mental wellbeing of older adults from both “variable-oriented” and “configuration-oriented” perspectives, thereby yielding richer and more robust research conclusions than could be achieved with a single method.

### 3.2 Scale variables and measurement items

The questionnaire used in this study was primarily designed on the basis of the Technology Acceptance Model (TAM), Self-Determination Theory (SDT), and theories related to wellbeing, and was further adapted and refined in accordance with the specific usage scenarios of smart older adults care services, as shown in Table 1. During the item development process, we first drew upon the core constructs of TAM, including “perceived usefulness,” “perceived ease of use,” and “behavioral intention to use.” Items were screened from established scales and relevant literature, and then localized and contextualized based on older adults’ actual experiences with smart older adults care, to ensure that the items were both theoretically oriented and easy for respondents to understand. Second, following the three basic psychological needs proposed in Self-Determination Theory—autonomy, competence, and relatedness—we designed measurement items corresponding to changes in older adults’ autonomous decision-making, sense of competence, and social connectedness after using AI-assisted tools, thereby capturing the impact of smart older adults care services on the satisfaction of their psychological needs. Third, drawing on theories of subjective wellbeing and psychological wellbeing, we constructed items along dimensions such as emotional state, life satisfaction, and psychological tranquility, in order to assess the relationship between the use of smart older adults care products and older adults’ overall wellbeing. With respect to the response format, the main part of the questionnaire adopts a five-point Likert scale. Each item provides five equally spaced response options, with scores from 1 to 5 representing “strongly disagree,” “disagree,” “neutral,” “agree,” and “strongly agree,” respectively. Higher scores on each item indicate a higher level of endorsement of, or a stronger experience with, the corresponding latent construct. Some items are reverse coded when necessary to control for response bias.

**Table 1:** Measurement scales and item sources.

Variable dimension	Explicit variable	Measurement item
Perceived ease of use (PEOU)	Operational ease	Learning to operate these systems or devices is easy for me.
	Interface clarity	The interfaces of these AI services are easy for me to understand and operate.
	Effort conservation	Becoming proficient in using these systems or devices does not require much effort on my part.
Perceived usefulness (PU)	Efficiency improvement	Using this system improves my efficiency in work or learning.
	Emotional support	Through AI (such as chatbots or online counseling), it is easier for me to obtain emotional support or psychological guidance.
	Health reminders	AI reminds me to take medication or seek medical attention, which aids in maintaining my physical and mental health.
Autonomy (AU)	Autonomous decision-making	Since using AI, I can make more decisions on my own in daily life without relying excessively on family members.
	Decision support	The suggestions provided by AI help me make my own choices regarding health or daily life.
	Independent management	Using AI makes me feel that I can still manage many matters independently.
Competence (CO)	Skill mastery	I feel that I have mastered the basic methods of using these AI products.
	Sense of achievement	Learning to use this device gives me a sense of achievement and makes me feel that I have not been left behind by the times.
	Self-efficacy	Using this product gives me more confidence in my ability to handle life’s problems.
Relatedness (RE)	Intimate interaction	Even when family members are not around, I can maintain intimate interactions with them through smart devices.
	Electronic companion	I feel that this smart device is like an “electronic companion” or an “old friend” to me.
	Social expansion	Interest groups or activities on AI platforms allow me to meet new friends or peers.
Mental wellbeing (MW)	Peace of mind	Recently, while using this product, I have felt pleasant and calm most of the time.
	Health assurance	I do not have excessive worry or anxiety about my physical condition and future life.
	Emotional stability	I am rarely troubled by long-term strong feelings of anxiety, irritability, or depression.

### 3.3 Data collection process

The data collection for this study was conducted from February to September 2025, utilizing a multi-channel approach that combined both online and offline surveys to ensure the breadth and representativeness of the sample. The questionnaire was primarily constructed based on the Technology Acceptance Model (TAM) and Self-Determination Theory (SDT), and was localized and optimized according to the practical application scenarios of smart older adult care services in China. The main part of the questionnaire employed a Likert 5-point scale, with options ranging from “1 = strongly disagree” to “5 = strongly agree,” covering core variables such as perceived ease of use, perceived usefulness, autonomy support, competence support, relatedness support, and mental wellbeing. All scales were adapted from well-established domestic and international questionnaires, and were appropriately adjusted after expert review and small-scale pilot testing to ensure scientific validity and applicability. To enhance the breadth and quality of the data, a mixed-methods approach combining online and offline surveys was adopted:

Online survey: Electronic questionnaires were designed and distributed via the “Wenjuanxing” platform, generating dedicated links and QR codes. The research team collaborated with local community older adult care service centers, universities for older adults, medical institutions, and smart older adult care platforms, inviting community workers and administrators of older adult care institutions to forward the questionnaire links and QR codes to social platforms commonly used by older adults.

Offline survey: Research team members visited partner community older adult care service centers, universities for older adults, neighborhood/street committees, and medical institutions to distribute paper questionnaires on-site, providing one-on-one guidance or assisted interviews for older adults in need. For those with poor eyesight or limited mobility, researchers assisted in completing the questionnaire through oral questioning and on-site recording, ensuring the completeness and accuracy of the data.

All participants were fully informed of the study’s purpose, content, and personal information protection measures before completing the questionnaire, and provided written or verbal informed consent. The study committed to strict confidentiality of all data, which would be used solely for academic research, ensuring participants’ anonymity and privacy rights. Through the above multi-channel and multi-method data collection process, a total of 491 questionnaires were collected. After excluding 73 invalid questionnaires, 418 valid samples were obtained, resulting in an effective response rate of 85.13%. Among the 418 valid samples, 189 were collected through online channels (45.22%) and 229 through offline channels (54.78%). This sample data provided a solid foundation for subsequent in-depth empirical testing using Structural Equation Modeling (SEM) and Fuzzy-Set Qualitative Comparative Analysis (fsQCA).

### 3.4 Descriptive statistical analysis of the sample

A total of 418 valid samples were ultimately included in the analysis for this study, and the demographic characteristics of the sample are shown in Table 2. The proportion of males and females was roughly balanced (56% male, 44% female), reflecting gender equilibrium in the sample. The age distribution was relatively comprehensive, with younger seniors aged 60–69 accounting for 61% of the sample, forming the main group; this cohort typically demonstrates higher acceptance and willingness to use new technologies. Those aged 70–79 accounted for 30%, while the oldest group, aged 80 and above, comprised 39 individuals (9%). Regarding the frequency of smart older adult care platform usage, 59% (*n* = 245) of older adults reported regular use (three times per week or more), 28% (*n* = 117) used the platforms occasionally (one to two times per month), and only 13% (*n* = 56) were first-time or infrequent users. The main types of platforms used were focused on health management, daily care, and emotional companionship. In terms of living arrangements, approximately 48% of older adults lived alone, 30% lived with a spouse, and 22% lived with their children, reflecting the diversity of family structures and living arrangements among older adults.

**Table 2:** Descriptive statistics of sample characteristics (*N* = 418).

Variable	Category	Frequency (Persons)	Percentage (%)
Gender	Male	234	56
	Female	184	44
Age	60–69 years old	254	61
	70–79 years old	125	30
	80 years old and above	39	9
Frequency of use on the smart senior care platform	Daily use (3 times or more per week)	245	59
	Occasional use (1–2 times per month)	117	28
	First-time or very infrequent use	56	13
Living arrangements	Living alone	200	48
	Living with spouse	125	30
	Living with children	93	22

## 4 Data analysis and results

### 4.1 Reliability and validity testing

To ensure the measurement quality of the scales, this study conducted reliability and validity tests on all major variables. The specific results are shown in Table 3. The Cronbach’s *α* coefficients for all constructs were above 0.75, exceeding the commonly used reliability threshold of 0.70, indicating good internal consistency for each scale. In terms of convergent validity, the average variance extracted (AVE) for each construct was greater than 0.50, demonstrating good convergent validity for all variables. The composite reliability (CR) values were also all above 0.78, further confirming the reliability and consistency of the scales. In summary, the measurement tools used in this study met the academic standards for reliability and validity (53), providing a solid measurement foundation for subsequent Structural Equation Modeling (SEM) and fsQCA analyses.

**Table 3:** Data reliability analysis table.

Construct	Item	Cronbach’s α	AVE	CR
Perceived ease of use (PEOU)	PEOU1	0.823	0.610	0.824
	PEOU2			
	PEOU3			
Perceived usefulness (PU)	PU1	0.860	0.674	0.861
	PU2			
	PU3			
Autonomy (AU)	AU1	0.778	0.549	0.784
	AU2			
	AU3			
Competence (CO)	CO1	0.824	0.555	0.789
	CO2			
	CO3			
Relatedness (RE)	RE1	0.785	0.609	0.823
	RE2			
	RE3			
Mental wellbeing (MW)	MW1	0.796	0.583	0.806
	MW2			
	MW3			

To assess whether the data were suitable for factor analysis, this study used SPSS 27.0 to conduct the KMO and Bartlett’s test of sphericity. The KMO value is 0.867, exceeding the excellent standard of 0.8 (54); the result of Bartlett’s test of sphericity was also significant (χ^2^ = 3337.206, *p* < 0.001). Overall, these two indicators demonstrate that the questionnaire data have good structural validity and meet the prerequisites for factor analysis, thus allowing for subsequent factor extraction.

In addition, since all data in this study were obtained from respondents’ self-reports and collected using a cross-sectional design, there may be a risk of common method bias (CMB). To examine this issue, the study adopted Harman’s single-factor test. All measurement items of the latent variables were entered into an exploratory factor analysis without rotation. The results showed that the first principal component accounted for 34.32% of the total variance, which is far below the critical threshold of 50%. Therefore, the data in this study are not subject to serious common method bias.

### 4.2 Goodness of fit indices of the structure model

To assess the structural validity of the questionnaire, this study conducted confirmatory factor analysis (CFA) to evaluate the model fit of the theoretical framework. The results showed that all key fit indices of the model performed excellently. Specifically, the relative chi-square value (CMIN/DF) was 1.72, which falls within the ideal range of 1–3, indicating a good overall model fit. In terms of absolute fit indices, the goodness-of-fit index (GFI) was 0.956, the adjusted goodness-of-fit index (AGFI) was 0.937, and the root mean square error of approximation (RMSEA) was 0.032, all meeting or exceeding commonly accepted statistical standards. Additionally, for incremental fit indices, the IFI was 0.984, NFI was 0.949, TLI was 0.979, and CFI was 0.984, all significantly higher than the excellent threshold of 0.90. In summary, all fit indices indicate that the questionnaire used in this study has good structural validity and that the model fits the actual data well.

### 4.3 Hypothesis testing

This study employed structural equation modeling (SEM) to test the theoretical hypotheses, and the results are presented in Table 3. All path coefficients reached statistical significance, indicating that each independent variable exerts a positive and significant impact on older adults’ mental wellbeing. Specifically, the standardized coefficient of perceived ease of use (PEOU) on mental wellbeing is 0.188 (*p* = 0.028), and that of perceived usefulness (PU) on mental wellbeing is 0.191 (*p* = 0.007); both are significant at the 0.05 level, thus supporting hypotheses H1 and H2. Autonomy (AU) has the greatest impact on mental wellbeing, with a standardized coefficient of 0.266 (*p* = 0.003) and the strongest level of significance, confirming hypothesis H3. The standardized coefficients of competence (CO) and relatedness (RE) on mental wellbeing are 0.211 (*p* = 0.008) and 0.215 (*p* = 0.014), respectively, both reaching statistical significance and supporting hypotheses H4 and H5. Taken together, perceived ease of use, perceived usefulness, autonomy, competence, and relatedness all exert significant positive effects on older adults’ mental wellbeing, suggesting that AI companion technologies jointly enhance older adults’ mental wellbeing through multidimensional mechanisms.

**Figure 3:**
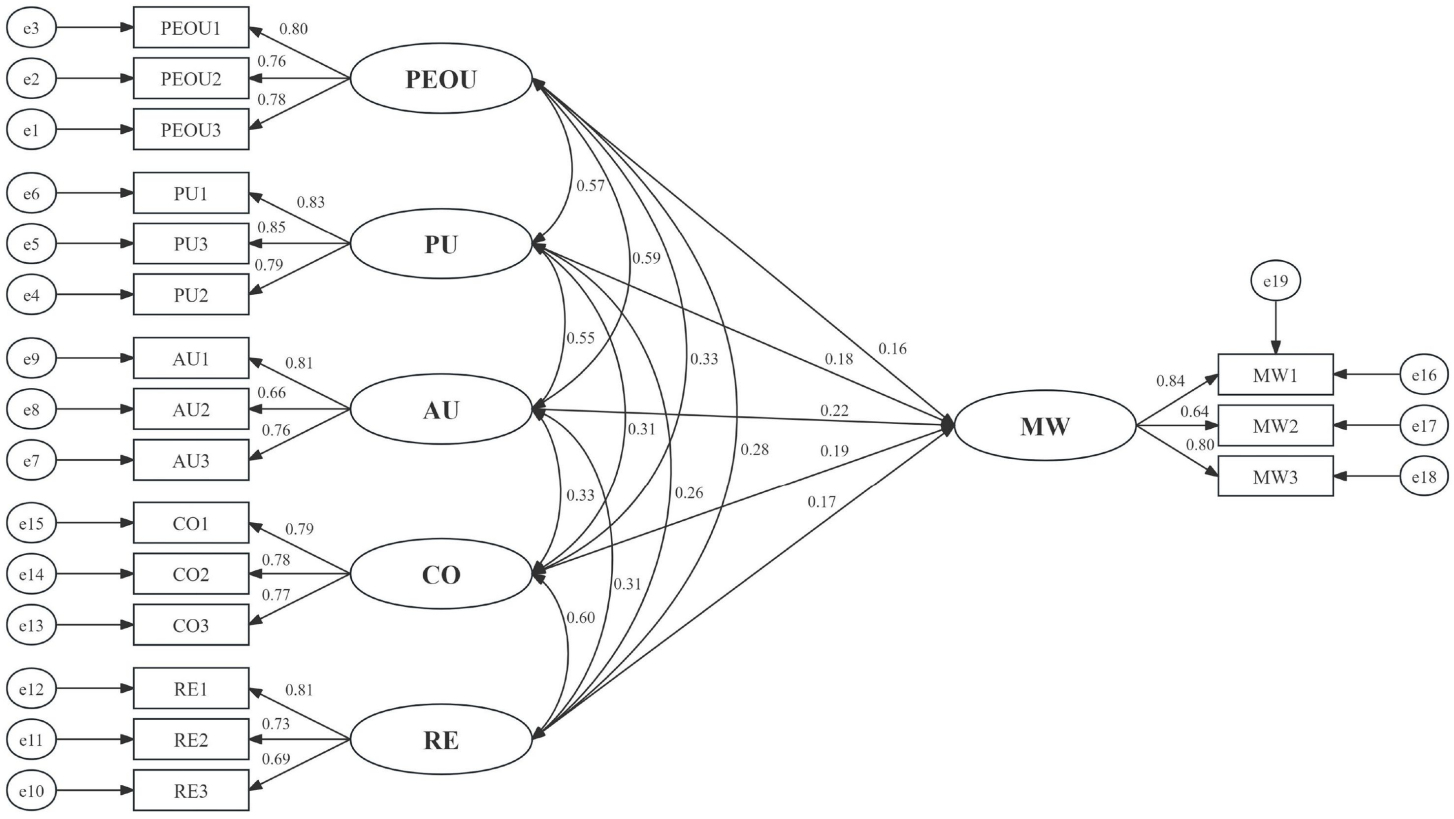
Research hypothesis diagram (All path coefficients in the table are standardized estimates, which facilitates comparison of the relative effect sizes across different variables and paths).

Finally, to evaluate the overall explanatory power of the theoretical model, we examined the squared multiple correlation coefficient (*R*^2^) of the endogenous latent variable. The results show that all antecedent and mediating variables in the model jointly explain 44.4% of the total variance in the ultimate dependent variable—older adults’ mental wellbeing (*R*^2^ = 0.444). According to Cohen’s criteria (55), this level of explained variance constitutes a large effect, which fully demonstrates that the integrated model constructed in this study has substantial practical significance and theoretical value for predicting and explaining older adults’ mental wellbeing (Figure 3 and Table 4).

**Table 4:** Hypothesis verification results.

Hypothesis		Standardized Estimate	S. E.	C. R.	*P*
H1	PEOU→MW	0.188	0.086	2.191	0.028
H2	PU → MW	0.191	0.070	2.713	0.007
H3	AU → MW	0.266	0.089	2.997	0.003
H4	CO → MW	0.211	0.080	2.639	0.008
H5	RE → MW	0.215	0.088	2.456	0.014

PEOU: Perceived Ease of Use; PU: Perceived Usefulness; AU: Autonomy; CO: Competence; RE: Relatedness; MW: Mental Wellbeing.

## 5 Fuzzy-set qualitative comparative analysis

### 5.1 Variable selection and calibration

In fuzzy-set qualitative comparative analysis (fsQCA), calibrating continuous variables is a prerequisite for conducting sufficiency analysis. Based on theoretical considerations and the actual distribution of the sample, and in order to objectively reflect the empirical distribution while avoiding the interference of extreme values, this study follows the classical approach of Fiss (56) and uses the 95th, 50th, and 5th percentiles of the sample data as the calibration anchors for full membership, the crossover point, and full non-membership, respectively.

As shown in Table 5, the fully affiliated point, crossover point, and fully unaffiliated point for the outcome variable mental wellbeing (MW) were set at 5, 4, and 2, respectively. This means that individuals scoring 5 are considered to have a high level of mental wellbeing, those scoring 2 or below are considered to have a very low level of mental wellbeing, and a score of 4 falls within the ambiguous range. Each condition variable (such as perceived ease of use, perceived usefulness, autonomy, competence, and relatedness) was also assigned corresponding calibration points based on its scale distribution. For example, the fully affiliated point for perceived ease of use (PEOU) was set at 5, the crossover point at 4, and the fully unaffiliated point at 2.33; for perceived usefulness (PU), the fully unaffiliated point was set at 1.67, reflecting sensitivity to the extreme states of the variable.

**Table 5:** Calibration points for each variable.

Variable		Fully affiliated points	Crossing points	Fully unaffiliated points
Outcome variable	MW	5	4	2
Condition variable	PEOU	5	4	2.33
	PU	5	4	1.67
	AU	5	4	2.33
	CO	5	3.67	2
	RE	5	3.67	2

PEOU: Perceived Ease of Use; PU: Perceived Usefulness; AU: Autonomy; CO: Competence; RE: Relatedness; MW: Mental Wellbeing.

This calibration method not only strictly adheres to the theoretical requirements of fsQCA, ensuring the scientific rigor of variable classification, but also enhances the discriminative power of variable measurement through refined threshold standards. This provides a solid foundation for the identification and interpretation of complex causal relationships in subsequent analyses, and demonstrates high academic innovation and empirical application value.

### 5.2 Analysis of necessary conditions

In fsQCA analysis, necessity testing is used to determine whether a single condition is a necessary prerequisite for the occurrence of the outcome variable (high level of mental wellbeing). Generally, when the consistency value exceeds 0.9, the condition can be considered a necessary condition for the outcome. As shown in Table 6, the consistency values for all condition variables [perceived ease of use (PEOU), perceived usefulness (PU), autonomy (AU), competence (CO), relatedness (RE) and their negations (~PEOU, ~PU, ~AU, ~CO, ~RE)] in relation to high mental wellbeing outcomes did not exceed 0.8. The highest was relatedness (RE) with a consistency of 0.759, and the lowest was the negation of autonomy (~AU) with a consistency of 0.533. The coverage indices also did not meet the necessity criteria. This indicates that neither individual high-level conditions nor their negations alone can constitute necessary conditions for high mental wellbeing outcomes; rather, different factors may work together in combination. Therefore, the research focus should shift to sufficiency analysis, examining how these conditions combine to jointly lead to the desired outcome.

**Table 6:** Necessity test.

Variable	High outcome variables	
	Consistency	Coverage
PEOU	0.740913	0.749951
~PEOU	0.539325	0.511637
PU	0.731211	0.710165
~PU	0.561299	0.554408
AU	0.758734	0.737614
~AU	0.532632	0.525573
CO	0.747689	0.702013
~CO	0.539232	0.551926
RE	0.759242	0.705487
~RE	0.550078	0.569517

In the table, “~” denotes the logical “NOT”; PEOU: Perceived Ease of Use; PU: Perceived Usefulness; AU: Autonomy; CO: Competence; RE: Relatedness; MW: Mental Wellbeing.

### 5.3 Sufficiency analysis

To further reveal the multiple pathways leading to high levels of mental wellbeing, this study used the fsQCA method to conduct configurational analysis of the condition variables. As shown in Table 7, four typical combinations of conditions (NH1–NH4) leading to high mental wellbeing (MW) were identified, with each pathway consisting of different core and peripheral conditions. In these configurations, perceived ease of use (PEOU), perceived usefulness (PU), autonomy (AU), competence (CO), and relatedness (RE) are combined in various ways to jointly enhance mental wellbeing. For example, in the NH1 pathway, PEOU, PU, and AU are all core conditions, indicating that when older adults highly perceive AI technology as easy to use, useful, and autonomy-enhancing, significant improvements in mental wellbeing can be achieved even if other conditions are not prominent. The NH2 pathway features PEOU and PU as core conditions, with CO and RE as peripheral conditions, highlighting the synergistic effect of technological attributes and certain psychological needs. The NH3 and NH4 pathways emphasize multiple supports for autonomy, competence, and relatedness, demonstrating the crucial role of psychological need satisfaction in promoting mental wellbeing.

**Table 7:** Resulting variable configuration table.

Conditional variable	NH1	NH2	NH3	NH4
PEOU	●	●	●	
PU	●	●		●
AU	●		●	●
CO		●	●	●
RE	⊗	●	●	●
Original coverage	0.352794	0.453901	0.465489	0.472807
Unique coverage	0.0947996	0.0331563	0.0447445	0.0520626
Consistency	0.884749	0.899829	0.907916	0.896776
Solution coverage	0.645508			
Solution consistency	0.847044			

“●” indicates the core condition is present; “●” indicates the peripheral condition is present; “⊗” indicates the core condition is absent; “⊗” indicates the peripheral condition is absent; a blank space indicates the presence or absence of the condition variable is irrelevant. PEOU: Perceived Ease of Use; PU: Perceived Usefulness; AU: Autonomy; CO: Competence; RE: Relatedness; MW: Mental Wellbeing.

The consistency of each configuration exceeds 0.88, with the highest reaching 0.91, indicating strong explanatory power of these pathways for high mental wellbeing outcomes. The raw coverage ranges from 0.35 to 0.47, and the unique coverage ranges from 0.03 to 0.09, suggesting that each configuration has a certain degree of uniqueness in explaining the outcome variable. Overall, the solution coverage is 0.646, indicating that these four pathways together explain 64.6% of high mental wellbeing cases, and the solution consistency is 0.847, further confirming the reliability of the explanatory results.

## 6 Discussion and conclusion

### 6.1 Discussion

This study systematically explores the mechanisms by which artificial intelligence (AI) companion technologies influence the mental wellbeing of older adults, utilizing Structural Equation Modeling (SEM) and Fuzzy-Set Qualitative Comparative Analysis (fsQCA). The results indicate that perceived ease of use (PEOU), perceived usefulness (PU), autonomy (AU), competence (CO), and relatedness (RE) all have significant positive effects on the mental wellbeing of older adults, and that these factors jointly promote mental wellbeing through multiple configurational pathways. This finding not only enriches the theoretical research in the field of smart older adult care but also provides scientific evidence for the practical application of AI technologies in promoting mental wellbeing among older adults.

The data clearly demonstrate that perceived ease of use and perceived usefulness are fundamental drivers for improving mental wellbeing. This corroborates the consensus in numerous human-computer interaction studies: for any technology—especially those targeting older adults who may experience digital anxiety—being “easy to use” and “useful” are the basic thresholds for acceptance (57). However, a further finding of this study is that psychological need variables represented by Self-Determination Theory (SDT)—particularly autonomy—exert an even greater influence than the two core variables of the Technology Acceptance Model (TAM). This sends a strong signal: the deeper impact of AI on the mental wellbeing of older adults stems more from its potential as a companion rather than merely a tool. When AI supports older adults in autonomous decision-making, enables them to experience competence in task management, and fosters emotional connections with the outside world, it transforms from a cold technological product into a social agent that nourishes basic psychological needs. This aligns with previous research that regards social robots as relational technologies (58), whose core value lies in emotional interaction and relationship simulation. By providing personalized services and interactive experiences, AI technologies can effectively enhance autonomy among older adults. For example, older adults can independently choose the content, frequency, and mode of interaction with AI, and this empowerment significantly increases their sense of control and self-efficacy (59). Fang et al., in their study on Chinese older adults using intelligent companion robots, found that those who could autonomously set robot functions and interaction modes had significantly higher levels of mental health than those who passively received technological services (60).

For older adults, technological barriers and learning costs are often the main obstacles to adopting new technologies (61). The results of this study show that if AI technologies can simplify operational processes, lower learning thresholds, and demonstrate clear practical value in daily life, they can significantly enhance the mental wellbeing of older adults. For instance, Pu et al., in a systematic review of the impact of social robots on the mental health of older adults, pointed out that AI devices with high ease of use and practical functions can effectively alleviate loneliness and anxiety, and improve life satisfaction among older adults (24). This finding is highly consistent with the results of this study, further validating the importance of perceived ease of use and perceived usefulness in promoting mental wellbeing in older adults. Competence and relatedness, as two other basic psychological needs in Self-Determination Theory, were also found in this study to have significant positive effects on the mental wellbeing of older adults. Competence refers to the sense of ability and achievement experienced by individuals in interacting with their environment; AI technologies, by providing cognitive training and health management functions, help older adults maintain cognitive vitality and self-care abilities, thereby enhancing their sense of competence (62). Relatedness emphasizes emotional connections with others or society; AI technologies, through voice interaction and emotion recognition, provide channels for emotional communication, partially alleviating social isolation and loneliness among older adults (51).

This multidimensional mechanism model illustrates that successful AI companion technology interventions must be an organic integration of technological design (TAM) and the fulfillment of psychological needs (SDT). A product that is “useful but difficult to use,” or “easy to use but fails to meet deep psychological needs,” is unlikely to have a lasting and profound positive impact on the mental wellbeing of older adults. The prominent role of “autonomy” in this study offers an important insight: when designing AI technologies for older adults, we should not only consider how to care for them, but also how to empower them. The ultimate goal of design should be to maximize users’ autonomy and sense of control, transforming them from passive recipients of care into active agents of their own lives.

### 6.2 Conclusion

With the accelerating process of global population aging, mental health issues among older adults have become increasingly prominent and are now a major focus of societal attention. As an emerging intelligent approach to older adult care, artificial intelligence (AI) technology has gradually become an important tool for enhancing the mental health of older adults due to its unique advantages in emotional support, cognitive stimulation, and social interaction. Based on empirical data, this study systematically analyzes the multidimensional mechanisms—such as autonomy, perceived ease of use, perceived usefulness, competence, and relatedness—through which AI technology affects the mental wellbeing of older adults, and conducts an in-depth discussion in conjunction with relevant theories and cutting-edge research. The findings reveal that the positive impact of AI technology on the mental health of older adults results from the synergistic effect of multiple mechanisms. Autonomy, perceived ease of use, perceived usefulness, competence, and relatedness interact and work together to form a systematic pathway for promoting mental wellbeing. This discovery enriches the theoretical framework of AI technology in the field of older adult mental health and provides new perspectives and ideas for subsequent research.

At the practical application level, the research results offer specific recommendations for the design and promotion of AI technology. Technology developers should place great emphasis on the autonomy needs of older adults by increasing product personalization and customization features; at the same time, they should simplify operational processes, enhance the ease of use and practicality of the technology, and lower the barriers for older adults to use it. In addition, attention should be paid to emotional interaction and social connection functions to help older adults establish and maintain positive social relationships. Governments and society should also intensify efforts to promote and popularize AI technology among the older adult population, advancing its widespread application within the older adult care service system.

Through the synergistic effect of multidimensional mechanisms, AI technology has significantly improved the mental wellbeing of older adults, with autonomy playing a particularly critical role. As technology continues to advance and application scenarios expand, AI technology is expected to become a vital force in promoting healthy aging and improving the quality of life for older adults. In the future, further optimization of technology design and improvement of service systems should be pursued to promote the global application of AI technology, bringing greater wellbeing to the older adult population.

### 6.3 Limitations and future directions

Despite this study’s systematic exploration of the multidimensional impacts of artificial intelligence (AI) technologies on older adults’ mental wellbeing and the meaningful findings obtained, several non-negligible limitations remain and should be addressed and refined in future research. First, this study is situated in the Chinese context, and the sample data are mainly drawn from older adults in specific regions of China. Compared with Western countries, China exhibits distinct characteristics in terms of state–citizen relations, family structure, intergenerational support, and sociocultural norms. Historically, most relevant studies have originated in Western countries, where assumptions are often grounded in individualistic social models, whereas China places greater emphasis on collectivism, family responsibility, and a government-led social support system. These differences may influence older adults’ acceptance of AI technologies, their motivations for use, and their mental wellbeing. Therefore, although this study provides empirical evidence for AI applications in the Chinese context, the applicability of its conclusions to other sociocultural settings requires further validation. Future research should more systematically compare the similarities and differences between Chinese and Western social models in the literature review and theoretical analysis, thereby clarifying the cultural boundaries of the present study’s applicability.

Second, the data in this study are mainly derived from older adults in specific regions, and the sample size and structure may exhibit certain biases with respect to gender, age, educational level, and economic status. Older adults in different regions and cultural contexts may show significant differences in their acceptance of AI technologies and psychological responses. Future studies should expand the sampling scope to include a more diverse older population, in order to enhance the representativeness and generalizability of the findings. In addition, the cross-sectional design of this study makes it difficult to reveal the long-term impacts and causal relationships between AI technologies and older adults’ mental wellbeing. Older adults’ mental health status and their attitudes toward AI technologies may change over time as their familiarity with these technologies increases. Future research could employ longitudinal designs to dynamically observe the sustained effects of AI technologies on older adults’ mental health and the evolving trends of these effects. Finally, the mechanisms through which psychological health is influenced warrant further exploration. This study mainly focuses on five dimensions—autonomy, perceived ease of use, perceived usefulness, competence, and relatedness—yet the determinants of older adults’ mental wellbeing are complex and multifaceted, and may also be shaped by the interplay of social support, family relationships, physical health, and other factors. Future work may construct more integrated theoretical models to systematically analyze the mental health effects of AI technologies under the synergistic influence of multiple factors.

Future research can be expanded and deepened in the following aspects. First, cross-cultural comparative studies can be conducted across different countries and cultural contexts to examine how cultural differences shape AI technology acceptance and its effects on mental wellbeing, thereby enhancing the international relevance and universality of the research. Second, multi-method and multidimensional measurement tools should be adopted. By integrating physiological indicators, behavioral data, and third-party assessments, researchers can improve the objectivity and scientific rigor of mental wellbeing measurements. At the same time, in-depth exploration of older adults’ real-life experiences and subjective perceptions of AI technologies can provide more targeted recommendations for technology optimization. Third, attention should be paid to the long-term effects and dynamic changes of AI technologies. Through longitudinal tracking studies, it is possible to systematically investigate the sustained influence of AI technologies on older adults’ mental health, uncover the dynamic evolution of the underlying mechanisms, and provide scientific evidence for policy formulation and service innovation.

In summary, AI technologies show broad application prospects and substantial potential in promoting older adults’ psychological wellbeing. With the continuous advancement of technology and the deepening of research, AI is expected to become an important pillar for improving older adults’ quality of life and achieving healthy aging. Future efforts should continuously monitor its developmental trajectory and actively explore innovative pathways, so as to bring greater wellbeing and care to the older population.

## Data Availability

The original contributions presented in the study are included in the article/supplementary material, further inquiries can be directed to the corresponding author/s.
